# Model representations of kerogen structures: An insight from density functional theory calculations and spectroscopic measurements

**DOI:** 10.1038/s41598-017-07310-9

**Published:** 2017-08-01

**Authors:** Philippe F. Weck, Eunja Kim, Yifeng Wang, Jessica N. Kruichak, Melissa M. Mills, Edward N. Matteo, Roland J.-M. Pellenq

**Affiliations:** 1 Sandia National Laboratories, P. O. Box 5800, Albuquerque, New Mexico 87185 USA; 2 Department of Physics and Astronomy, University of Nevada Las Vegas, 4505 Maryland Parkway, Las Vegas, NV 89154 USA; 3 MultiScale Materials Science for Energy and Environment (MSE2), The Joint CNRS-MIT Laboratory, UMI CNRS 3466, Massachusetts Institute of Technology, Cambridge, Massachusetts 02139 USA; 4 Department of Civil and Environmental Engineering, Massachusetts Institute of Technology, Cambridge, Massachusetts 02139 USA; 5 CINaM-Aix Marseille Universite-CNRS, Campus de Luminy, 13288 Marseille Cedex 09, France

## Abstract

Molecular structures of kerogen control hydrocarbon production in unconventional reservoirs. Significant progress has been made in developing model representations of various kerogen structures. These models have been widely used for the prediction of gas adsorption and migration in shale matrix. However, using density functional perturbation theory (DFPT) calculations and vibrational spectroscopic measurements, we here show that a large gap may still remain between the existing model representations and actual kerogen structures, therefore calling for new model development. Using DFPT, we calculated Fourier transform infrared (FTIR) spectra for six most widely used kerogen structure models. The computed spectra were then systematically compared to the FTIR absorption spectra collected for kerogen samples isolated from Mancos, Woodford and Marcellus formations representing a wide range of kerogen origin and maturation conditions. Limited agreement between the model predictions and the measurements highlights that the existing kerogen models may still miss some key features in structural representation. A combination of DFPT calculations with spectroscopic measurements may provide a useful diagnostic tool for assessing the adequacy of a proposed structural model as well as for future model development. This approach may eventually help develop comprehensive infrared (IR)-fingerprints for tracing kerogen evolution.

## Introduction

Kerogen is a high-molecular weight, carbonaceous polymer material resulting from the condensation of organic residues in sedimentary rocks; such organic constituent is insoluble either in aqueous solvents or in common organic solvents^1^. In addition to its carbon skeleton, kerogen is also predominantly made of hydrogen and oxygen, as well as residual nitrogen and sulfur. Although kerogen plays a central role in hydrocarbon (i.e., crude oil and natural gas) production from source rocks in geologic environments^2^, the crucial interplay between its complex nanoscale structure and its properties has only been revealed in recent years^3–7^. A detailed understanding of the various kerogen structures associated with different oil- or gas-prone kerogen types and maturity is particularly important to understanding nanofluidic processes underlying crude oil and natural gas extraction^7^.

During its diagenesis, catagenesis, and metagenesis maturation stages, kerogen undergoes successive transformations concomitant with a gradual increase in *sp* ^2^/*sp* ^3^ carbon hybridization ratio^3, 5^ before eventually reaching full condensation of its polynuclear aromatic units to form *sp* ^2^-hybridized graphitic structures as components of a complex network of carbon nanopores. Existing model structures of kerogen were generally constrained from elemental analyses and functional group data obtained from X-ray photoelectron spectroscopy (XPS) and ^13^C nuclear magnetic resonance analyses (NMR)^4^. Recently, Bousige *et al*. developed kerogen structure models using a molecular dynamics-hybrid reverse Monte-Carlo (MD-HRMC) method, which minimizes the configurational energy while constraining possible molecular configurations from the pair distribution function obtained from inelastic neutron scattering measurements^5^.

Direct comparison between vibrational properties measured by neutron, Raman or infrared spectroscopies and spectra simulated from classical or quantum mechanical methods has proven to be a powerful tool for model validation^5, 8^. In this study, infrared signatures for a variety of generic kerogen models proposed recently^4, 5^ were calculated within the framework of density functional perturbation theory (DFPT) and compared to Fourier transform infrared (FTIR) spectra collected from kerogen samples with different types and maturity. Computed infrared (IR) signatures were also compared to generalized phonon densities of states (GDOS) obtained from recent inelastic neutron scattering experiments for various kerogen samples^5^. An attempt is made in this study to critically assess the adequacy of existing model representations of kerogen structures, using characteristic IR signatures as a diagnostic tool. We will show that a combination of DFPT calculations with vibrational spectroscopic measurements, especially FTIR measurements, can be a useful tool for developing more realistic models for kerogen structure representation. This approach may eventually lead to the development of a comprehensive IR-fingerprints for tracing kerogen evolution. Note that IR spectroscopy was successfully used in previous investigations to differentiate crude oil from residual fuel oil samples^9, 10^.

## Results and Discussion

**Figure 1:**
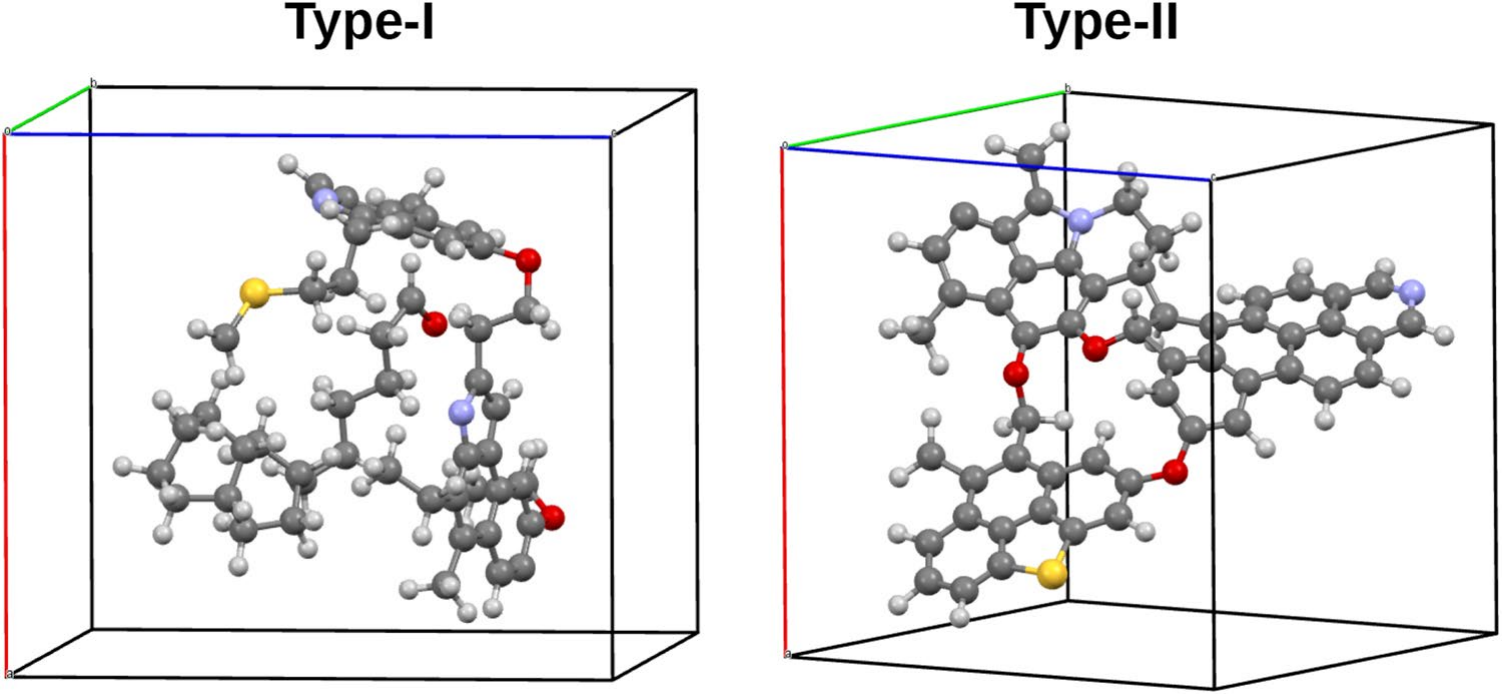
Structures of 3D-periodic type-I (left) and type-II (right) kerogen models used in DFT/DFPT calculations at the GGA/PBE level of theory. The simulation cells are indicated by solid lines. Color legend: grey, C; white, H; purple, N; red, O; yellow, S.

The 3D-periodic kerogen structures initially considered in this study were based on generic molecular fragments proposed by Ungerer *et al*.^4^ to represent kerogen structures with lacustrine (type I) and marine (type II) depositional origin. Type I kerogen oil shales are typically the most promising deposits for conventional oil retorting and type II kerogen is found in numerous oil shale deposits. For that reason, the focus of this study will be mainly on type-I and -II kerogen. The models utilized (cf. Fig. 1) contained 118 atoms (type-I) and 89 atoms (type-II) per simulation cell. In terms of oxygen-to-carbon (O/C) and hydrogen-to-carbon (H/C) atomic ratios, these type I (H/C = 1.29; O/C = 0.06) and type II (H/C = 0.57; O/C = 0.06) models correspond to kerogen in upper- and lower-left quadrants of the van Krevelen diagram, mapping H/C as a function of O/C to represent the chemical evolution of kerogen^11, 12^. Kerogen maturation over geologic times is accompanied by a decrease of H/C and O/C atomic ratios. In terms of vitrinite reflectance *R*_0_, another common maturity indicator, these type I and II models correspond to ~0.6 and ~1.5%*R*_0_, respectively; higher values of %*R*_0_ indicate higher maturity of the samples.

**Figure 2:**
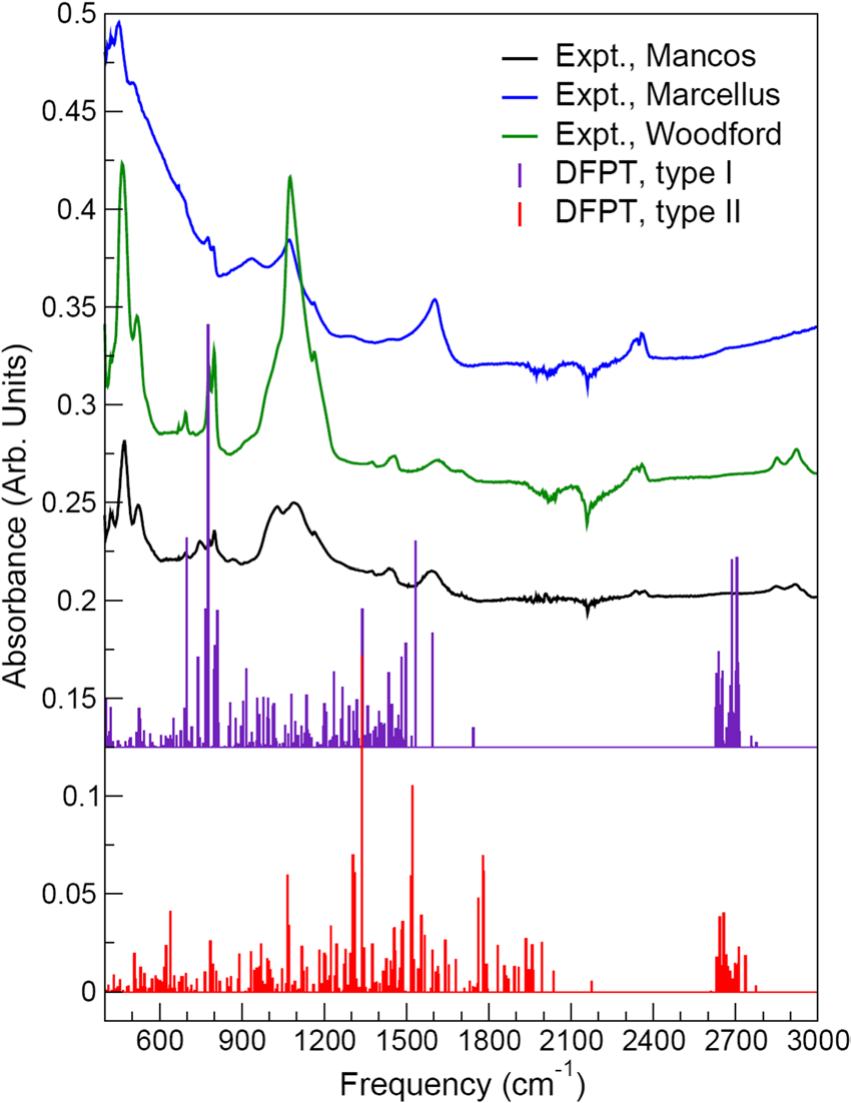
Infrared spectra simulated from density functional perturbation theory (DFPT) at the GGA/PBE level for the type-I and type-II models shown in Fig. 1 and Fourier transform infrared spectra collected for Mancos, Marcellus and Woodford kerogen samples.

Typical bands of interest for spectral signatures of kerogen include mainly the aliphatic band (2800–3000 cm^−1^), aromatic bands (700–900 cm^−1^, ~1600 cm^−1^, and 3000–3100 cm^−1^) and carbonyl and carboxyl absorption band (~1700 cm^−1^). Results from DFPT linear response and Born effective charge (BEC) calculations carried out at the generalized gradient approximation (GGA) level of theory with the parameterization of Perdew, Burke, and Ernzerhof (PBE) for those type-I and type-II models are shown in Fig. 2, along with FTIR spectra collected for Woodford, Marcellus, and Mancos kerogen samples. Based on the measurements of hydrogen index (HC/TOC), oxygen index (CO_2_/TOC) and vitinite reflectance, we have determined that the Woodford shale organic content is overwhelmingly composed of type-II kerogen, with a vitrinite reflectance value of 0.63%*R*_0_. The Mancos sample is a gas-prone, type-III kerogen (humic origin), with a vitrinite reflectance of 0.65%*R*_0_. The Marcellus sample is composed of mainly Type-II kerogen, with an estimated vitrinite reflectance value of 2.51%*R*_0_. While the simulated frequencies and intensities vary in general from experimental values, agreement between DFPT results and experimental data is generally slightly better using the type-I model than with its type-II counterpart. In particular, compared to all experimentally characterized samples, the type-II model tends to overestimate IR absorbance in the 1200–1400 cm^−1^ and 1700–2200 cm^−1^ ranges, while the type-I model simulations feature intensity ratios more in line with experiments. Therefore, details of the present vibrational analysis given below will focus on the type-I model, although results for the type-II model will also be discussed.

In the 2600–2800 cm^−1^ range, both type-I and type-II models display a similar double-peak structure characteristic of C–H stretch, with the asymmetric and symmetric C–H stretches corresponding mainly to the highest and lowest-frequency peaks, respectively; C–H stretches from carbon rings are also in this frequency domain. The simulated frequencies are redshifted compared to measured symmetric and asymmetric C–H stretches centered around ~2850 and ~2920 cm^−1^, respectively, for the Woodford and Mancos samples. This can be ascribed in part to the inability of standard DFT/DFPT to describe accurately long-range van der Waals interactions resulting from dynamical correlations between fluctuating charge distributions and to the fact that the type-I and type-II models used in this study might be more representative of isolated molecular fragments than confined, entangled molecular structures forming kerogen bulk; in the latter case, a blueshift of the simulated C–H stretch frequencies with respect to the present simulations would be expected. It is also worth noting that the type-II model used here still has significant IR absorbance in the 2600–2800 cm^−1^ range, suggesting that the number of C–H bonds in the model might not be representative of very mature samples with high level of carbon condensation such as Marcellus.

For the type-I model, the highest-frequency mode for ring C–C bond stretching is at 1743 cm^−1^ (2174 cm^−1^ in the type-II model), followed by an intense series of peaks below ~1595 cm^−1^ originating from combinations of ring C–C bond vibration and C–H twisting. Series of H–C–H scissoring and/or rocking modes are predicted around 1430–1495 cm^−1^. These ring C–C bond vibration/C–H twisting and H–C–H scissoring and/or rocking modes are most likely responsible for the FTIR peaks centered at ~1600 and 1450 cm^−1^, respectively. The fact that the peak around ~1600 cm^−1^ subsists for all levels of maturity of the samples, while it becomes sharper and experiences a slight redshift with kerogen maturity, confirms that it is due to ring C–C bond vibration (and becomes more pronounced and more uniform with carbon condensation into larger, regular 2D carbon structures). Since the peak centered around ~1450 cm^−1^ for Woodford and Mancos samples tends to fade away in the Marcellus sample, this also indicates that significant H–C–H scissoring and/or rocking modes have been suppressed as a result of dehydrogenation and condensation in the most mature samples. A smaller peak observed around ~1370 cm^−1^ for Woodford and Mancos samples and absent for the Marcellus sample is assigned, based on our type-I calculations, to combinations of CH_3_ “umbrella” modes and H–C–H wagging modes. Vibrational modes dominating in the ~960–1250 cm^−1^ range are complex combinations of H–C–H twisting, wagging and rocking, C–H and CH_3_ wagging, and ring C–C stretch. In particular, calculations suggest that the double-peak structure around ~1030 and ~1090 cm^−1^ for the Mancos sample (and to some extent the Woodford sample featuring a broad peak centered around ~1070 cm^−1^) stems predominantly from C–C stretching in hydrocarbon chains and C–C stretching in carbon rings; this would explain why the former peak fades away as hydrocarbon chains disappear in the mature Marcellus sample, while the latter peak subsists in mature samples, although narrower and slightly redshifted as a result of kerogen condensation. The sharp peaks in the vicinity of ~780 and ~800 cm^−1^ are assigned to C–C–C ring bending modes and C–O stretching modes in chains and rings, respectively, and the peak near ~740 cm^−1^ in the Mancos sample is assigned to ring C–N/C–C vibrations. The peak near ~695 cm^−1^ in both Woodford and Mancos samples is also assigned to C–O stretching modes in chains; this peak tends to disappear in more mature samples. At lower frequencies, a number of complex combinations of carbon skeletal modes from rings and chains contribute to the IR absorbance. The peak observed at ~520 cm^−1^ in both Woodford and Mancos samples (as well as in the Marcellus sample to some extent) originates from C–S stretching. The modes below this frequency are due essentially to complex, collective ring vibrational modes.

**Figure 3:**
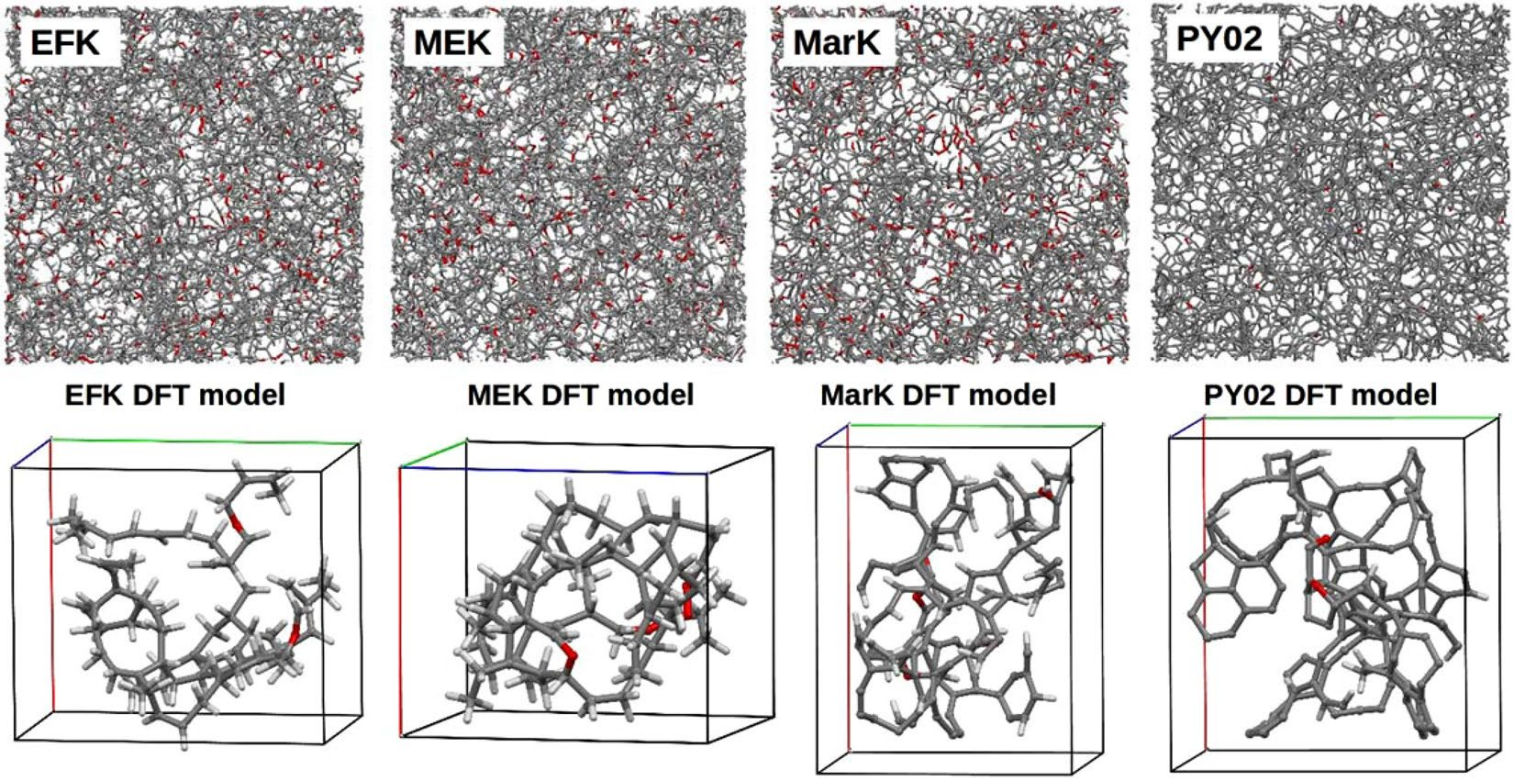
Structures of the EFK, MEK, MarK and PY02 models (Top; cubic box size of 50 × 50 × 50 Å^3^); representative 3D-periodic portions of EFK, MEK, MarK and PY02 models used in the present DFT/DFPT calculations at the GGA/PBE level (Bottom); simulations cells are indicated by solid lines). Color legend: grey, C; white, H; red, O.

**Figure 4:**
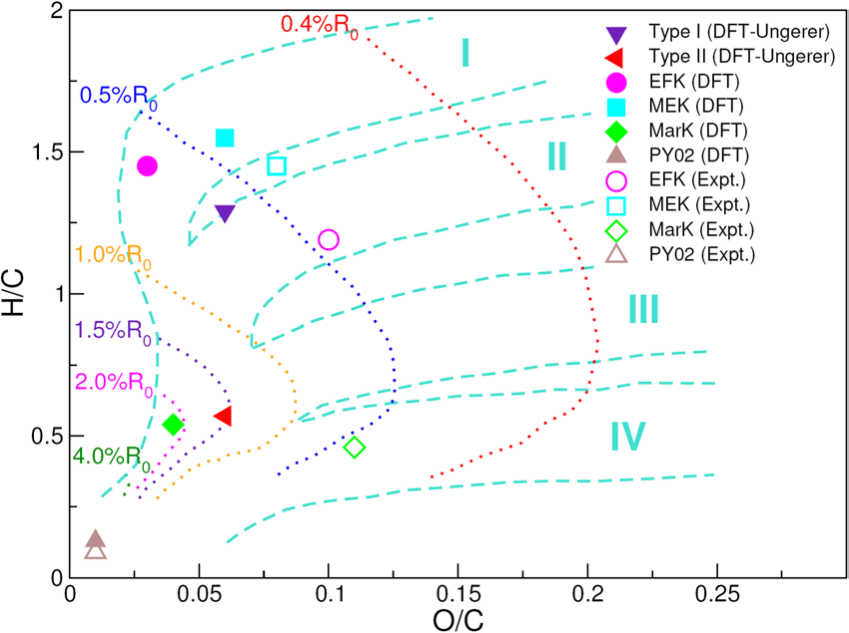
van Krevelen diagram displaying H/C versus O/C ratios for kerogen structures of the sophisticated EFK, MEK, MarK and PY02 models from Bousige *et al*. (ref. 5) and their simplified variants used in the present DFPT calculations, as well as the type-I and -II structures proposed by Ungerer *et al*. (ref. 4). Isovalue contour lines of the vitrinite reflectance *R*_0_, another common maturity indicator, are also represented, along with typical domains (cyan) for kerogen samples with different depositional origins (types I to IV). Maturation increases with decreasing H/C and O/C ratios.

In addition to the type-I and -II models proposed by Ungerer *et al*.^4^, complex kerogen structures based on the sophisticated EFK, MEK, MarK and PY02 models (cf. Fig. 3) recently proposed by Bousige and co-workers^5^ were also used to simulate IR spectra. These four molecular models with a density of 1.2 g/cm^3^ were built to represent an immature type-II kerogen from the carbonate-rich Eagle Ford Play (EFK; H/C = 1.19; O/C = 0.10; 0.65%*R*_0_), an immature sulphur-rich type-II kerogen from the Middle East (MEK; H/C = 1.45; O/C = 0.08; 0.55%*R*_0_), a mature type II kerogen from the clay-rich Marcellus Play (MarK; H/C = 0.46; O/C = 0.11; 2.20%*R*_0_), and a shungite from Russia (PY02; H/C = 0.09; O/C = 0.01) sharing many features with excessively mature kerogen^5^. The 3D-periodic portions of EFK, MEK, MarK and PY02 models used in the present DFT/DFPT calculations are also displayed. These smaller models have somewhat different H/C and O/C ratios (see Fig. 4) and lower densities than the samples characterized experimentally (*i.e*., EFK: H/C = 1.45, O/C = 0.03, ρ = 0.7 g/cm^3^; MEK: H/C = 1.55, O/C = 0.06, ρ = 0.8 g/cm^3^; MarK: H/C = 0.54, O/C = 0.04, ρ = 0.6 g/cm^3^; PY02: H/C = 0.13, O/C = 0.01, ρ = 0.5 g/cm^3^). The models used in DFT/DFPT calculations (cf. Fig. 3) contained 174 (EFK), 165 (MEK) and 119 (MarK and PY02) atoms per simulation cell. Experimental characteristics of the EFK, MEK and MarK kerogen samples and of the Mancos, Woodford and Marcellus kerogen samples discussed above are summarized in Table 1.

**Table 1:** Experimental characteristics of the Mancos, Woodford, Marcellus, EFK, MEK and MarK kerogen samples^(1)^.

Parameters	Mancos	Woodford	Marcellus	EFK	MEK	MarK
Total organic content TOC (wt%)	1.08	5.74	3.74	7.3	15.4	5.1
Rock eval S1 (mg HC/g)	0.37	0.68	0.14	2.02	5.21	0.27
Rock eval S2 (mg HC/g)	1.41	35.02	0.41	45.52	111.34	0.14
Rock eval S3 (mg HC/g)	0.05	0.41	0.08	0.99	1.95	0.86
Rock eval *T*_max_ (C°)	434	433	537	419	410	—
Vitrinite reflectance *R*_0_ (%)	0.65^(2)^	0.63^(2)^	2.51^(2)^	0.65	0.55	2.20
Hydrogen index (S2 × 100/TOC)	131	610	11	623	722	3
Oxygen index (S3 × 100/TOC)	5	7	2	13	13	17

^(1)^EFK, MEK and MarK characterization from ref. (5). ^(2^^)^Calculated with *R*_0_ = [(0.018 * *T*_max_) − 7.16].

**Figure 5:**
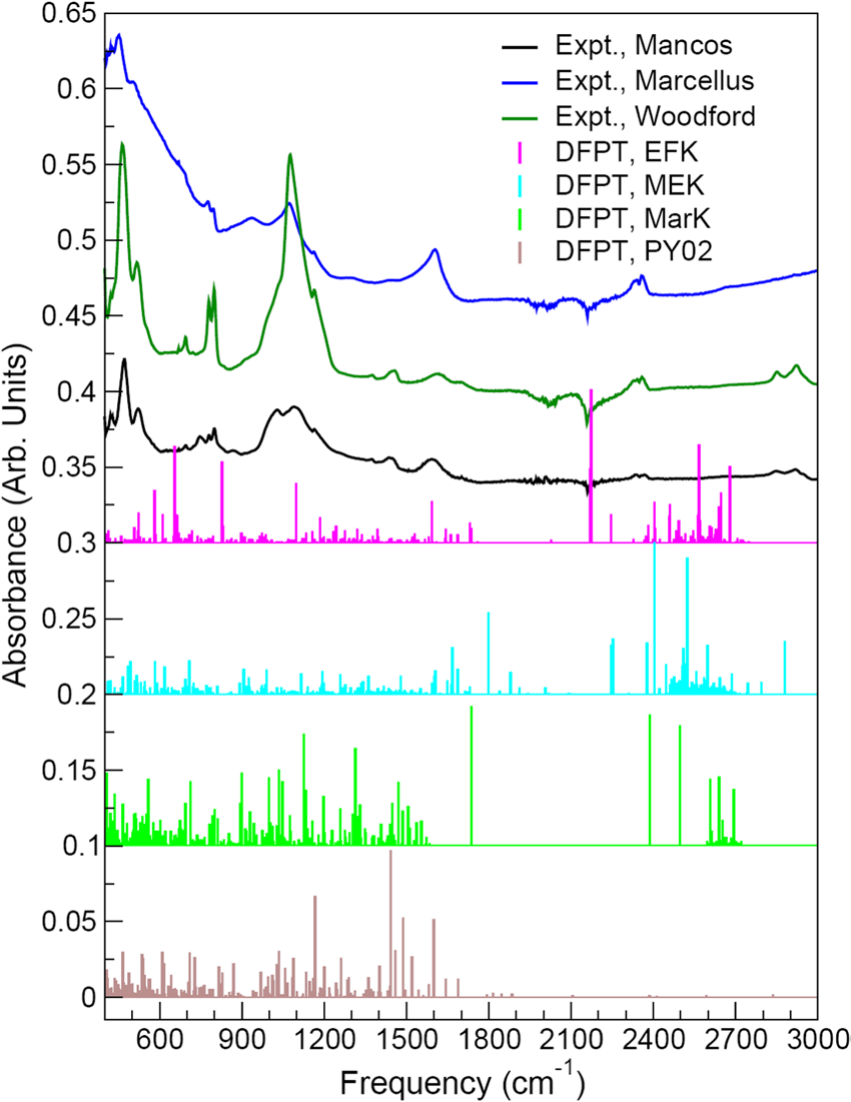
Infrared spectra simulated from density functional perturbation theory (DFPT) at the GGA/PBE level for representative portions of the EFK, MEK, MarK and PY02 models displayed in Fig. 3 and Fourier transform infrared spectra collected for Mancos, Marcellus and Woodford kerogen samples.

**Figure 6:**
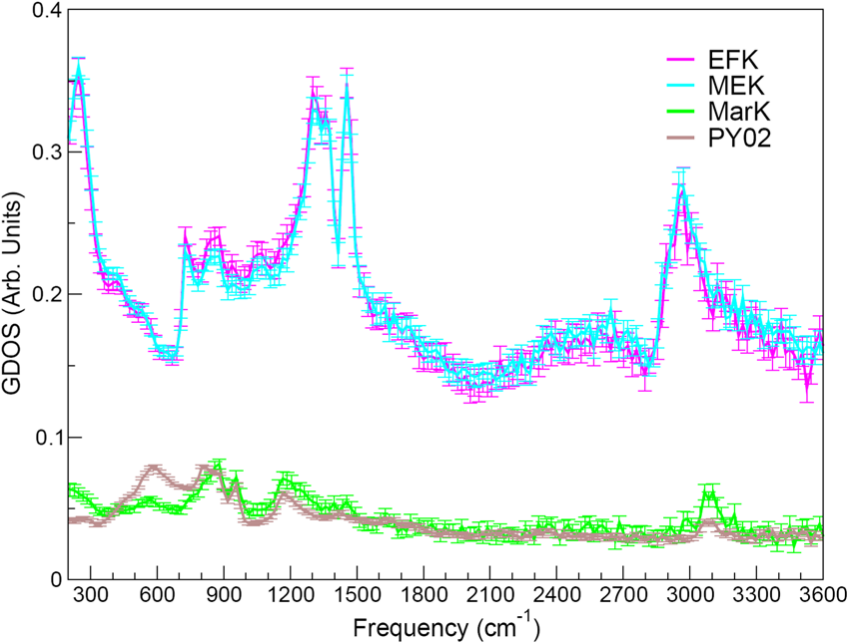
Generalized phonon densities of states (GDOS) of the EFK, MEK, MarK and PY02 samples from inelastic neutron scattering experiments (ref. 5). Error bars are computed from the square root of the neutron count.

Figure 5 shows the infrared spectra simulated using DFPT for the representative 3D-periodic portions of the EFK, MEK, MarK and PY02 models depicted in Fig. 3, along with the FTIR spectra of the Mancos, Marcellus and Woodford kerogen samples. A detailed analysis of the generalized phonon densities of states was carried out for the EFK, MEK, MarK and PY02 samples by Bousige *et al*. using force-field molecular dynamics simulations and inelastic neutron scattering measurements^5^. All vibrational eigenmodes are accessible by neutron spectroscopy, unlike in IR spectroscopy where selections rules apply. Therefore, the complex vibrational characteristics of these models will be only discussed succinctly hereafter. Nevertheless, as shown in ref. 5, the GDOS measured by neutron spectroscopy (cf. Fig. 6) are dominated by vibrational modes of hydrogen atoms forming bonds with carbon by *s-sp* ^2^ or *s-sp* ^3^ overlap. While such neutron spectroscopy measurements probing C–H bonding are useful to estimate maturation in terms of *sp* ^2^/*sp* ^3^ or H/C ratios, IR spectroscopy can also provide valuable information in terms of C–O, C– S, C–N, C–C and complex carbon-backbone vibrational modes, as discussed above for the analysis of type-I and -II models of Ungerer *et al*.^4^. Such information is crucial for constraining functional group distributions and their chemical bonding environments in kerogen.

Note that models used in this study cover a wide range of parameter space in the van Krevelen diagram (Fig. 4). We would expect that the three kerogen samples we studied should be reasonably represented by some of these models. However, the comparison of the calculated with the measured FTIR spectra (Figs 2 and 5) shows only a limited agreement between model predictions and experimental measurements, thus highlighting a possible deficiency of the existing models in the representation of actual kerogen structures, especially for functional group distributions in the material. Previous work shows that nanometer-scale pore structures and pore surface functional groups are the two most important attributes controlling hydrocarbon sorption and release^7, 13^ As shown in Fig. 6, existing models appear to have a reasonable representation of hydrogen pairing distribution as well as pore size distribution^5^ but have difficulty in capturing some key characteristics of the measured FTIR spectra, especially related to C–O, C–S, C–N, C–C vibrational modes. The work on other natural carbon materials indicates a critical role of surface functional groups in chemical sorption and desorption. For example, metal sorption on activated carbon has been found to highly depend on surface functional groups^14^. Surface functional groups can also modify the surface wettability of carbon materials^15^. It is anticipated that functional groups on kerogen pore surface may directly control hydrocarbon disposition and release in unconventional reservoirs. A similar mechanism may also play a critical role in regulating metal release from shale matrix in unconventional oil/gas production, an issue of a great environmental concern^16^. Therefore, development of new structural representation models that can better capture both nanoscale pore structures and surface functional groups of actual kerogen is highly desirable. Our work shows that a combination of DFPT calculations with spectroscopic measurements may provide a useful tool for future model development.

We acknowledge that the comparison made above between model predictions and experimental data is relatively rough. Kerogen is known for its structural heterogeneities^1^. A possible improvement to the existing comparison could perform DFPT simulations for different possible model structures and then use an averaged FTIR spectrum for comparison. It is expected that a large set of different kerogen model structures would be required to obtain a realistic and statistically-meaningful ensemble of kerogen properties to represent samples characterized experimentally. In this sense, it would be helpful to develop a limited number of end-member model structures such that an actual kerogen structure can represented by a combination of those end-member model structures. The existing classification of kerogen using the van Krevelen diagram focuses mainly on the origin of the carbon materials and their compositional evolution during thermal cracking, with less attention on an associated structural change (in particular, a change in surface functional group distribution). Given the structural heterogeneities, it would be very difficult, if not impossible, to represent actual kerogen structures with one or a definite set of “representative” model structures. The proposed end-member structure concept will help circumvent this difficulty, in which an actual kerogen structure will be described with a probabilistic distribution of the end members. This approach may also lead us to develop a comprehensive library of infrared fingerprints for tracing kerogen structural evolution.

## Methods

First-principles total energy calculations were carried out using DFT, as implemented in the Vienna *ab initio* simulation package^17^ (VASP). The exchange-correlation energy was calculated using GGA with the PBE parameterization^18^ (PBE). Such standard functionals were found in previous studies to correctly describe the geometric parameters and vibrational properties of a variety of carbonaceous structures^6, 19–21^. The interaction between valence electrons and ionic cores was described by the projector augmented wave (PAW) method^22, 23^. The C(2 *s* ^2^,2*p* ^2^), N(2 *s* ^2^,2*p* ^3^), O(2 *s* ^2^,2*p* ^4^) and S(3 *s* ^2^,3*p* ^4^) electrons were treated explicitly as valence electrons in the Kohn-Sham (KS) equations and the remaining core electrons together with the nuclei were represented by PAW pseudopotentials. The KS equation was solved using the blocked Davidson iterative matrix diagonalization scheme^24^. The plane-wave cutoff energy for the electronic wavefunctions was set to 500 eV, ensuring the total energy of the system to be converged to within 1 meV/atom. Electronic relaxation was performed with the residual minimization method direct inversion in the iterative subspace (RMM-DIIS), preconditioned with residuum-minimization. A periodic unit cell approach was used in the calculations. Kerogen models based on the structures reported recently by Ungerer *et al*.^4^ and Bousige *et al*.^5^ with different depositional origins and maturity levels were used in the calculations. Integrations in the Brillouin zone were carried out at the Γ point. Using these structures, DFPT linear response calculations were carried out at the GGA/PBE level of theory with VASP to determine the vibrational frequencies and associated intensities. The latter were computed based on the Born effective charges tensor, which corresponds to the change in atoms polarizabilities with respect to an external electric field. Compared to simple finite-displacement approaches typically used in DFT methods to describe vibrational properties of many-particle systems, DFPT-based methods can be regarded as considerably more effective since additional physical properties can be derived from the total energy with respect to perturbations^25^. We extensively tested the accuracy of DFPT approaches, with and without long-range corrections for van der Waals interaction, in previous studies^8, 26–31^.

Benchmark test calculations were carried out using VASP DFPT for simple molecules in the gas phase. For the linear CO_2_ molecule (*D*_4h_ group), the IR-active C–O bending (*E*_u_) and asymmetric stretch modes (*A*_2u_) were predicted to be at 631 and 2362 cm^−1^, respectively, i.e., in fair agreement with the measured values of 670 and 2350 cm^−1^ ^32^. The computed IR-active C–H modes for the CH_4_ molecule (*T*_d_ group) were 1353 and 2851 cm^−1^ for the bending (*T*_2_) and asymmetric stretch modes (*T*_2_), respectively. These results are comparable to the experimental values of 1306 and 3019 cm^−1^ for gaseous CH_4_^32^. Some of the discrepancies between DFPT and experimental FTIR results might stem in part from the fact that DFPT/DFT methods do not accurately predict the long-range van der Waals interactions.

Functional groups were identified and analyzed using FTIR spectroscopy on kerogen extracted from shale samples of the Mancos (Utha, USA), Woodford (Oklahoma, USA) and Marcellus (Pennsylvania, USA) formations obtained from TerraTek Inc (Schlumberger). The kerogens were lightly crushed using a mortar and pestle, then analyzed. Powdered samples were placed directly on the attenuated total reflection (ATR) attachment-Smart Orbit Diamond (3000–200 cm^−1^ band) for analysis. Spectra were collected using a Thermo Nicolet 380 FTIR spectrometer and OMNIC software suite. Scans were done on each sample at a resolution of 4 cm^−1^, with absorbance spectra ranging from 4000 to 400 cm^−1^. Blank readings were used between every samples.
